# Editorial: Resilience: Life Events, Trajectories and the Brain

**DOI:** 10.3389/fpsyt.2021.645687

**Published:** 2021-02-17

**Authors:** Jutta Lindert, Oliver Tüscher

**Affiliations:** ^1^Department of Social Work and Health, University of Applied Sciences, Emden/Leer, Emden, Germany; ^2^Department of Psychiatry and Psychotherapy, Leibniz Institute for Resilience Research (LIR), Mainz, Germany

**Keywords:** resilience, life events, trauma, trajectories, gender, neurobiology, longitudinal studies

Given the prevalence of stressful events and their impact on individuals, families, communities and societies, promoting and supporting resilience is a global societal need and challenge at the same time. There is considerable variation in the way resilience is currently being understood, defined, and measured both within animal and human studies. Resilience has been defined as either characteristic of the individual or as a dynamic cultural pluralistic interactional process. Based on evolving research the concept of resilience has significantly changed from a trait-oriented to a dynamic outcome, which draws on concepts of life-course epidemiology, trans-diagnostic psychiatry, developmental psychology, emotion research, and cognitive neuroscience. We initiated this Research Topic by proposing that resilience is dynamic interactional process, which includes distal and proximal protective and risk factors/mechanisms. In parallel with articles published in this Research Topic, recent research has advanced our knowledge of resilience highlighting that resilience is an interactive multidimensional process. In light of this contemporary research, the present Research Topic on “Resilience: Life Events, Trajectories and the Brain” in Frontiers in Psychiatry makes a contribution with articles comprising original research, reviews, and theoretical insights.

Three articles considered resilience in the context of early life events. Up to half of children and adolescents in Western societies experience at least one type of early life adversity. Accordingly, these children and youth might be at risk of developing stress-related psychopathologies. In a systematic review, Fritz et al. found empirical evidence for 20 resilience factors (individual level, family resilience factors, and community factors). This highlights the importance of considering resilience factors on different levels of analyses and suggests that resilience factors systems rather than single resilience factors should be studied (Fritz et al.). Another insightful review by Agorastos et al. adds knowledge on human stress system modifications due to early life stress. This review discusses evidence from mainly human research on the 10 most acknowledged neurobiological allostatic pathways exerting enduring adverse effects of early life adversity decades later (hypothalamic-pituitary-adrenal axis, autonomic nervous system, immune system and inflammation, oxidative stress, cardiovascular system, gut microbiome, sleep and circadian system, genetics, epigenetics, structural, and functional brain correlates). Understanding the effects of dysregulated interconnection between all neural systems involved provides new insights into the pathophysiologic trajectories that link early life stress during developmental stages of childhood and adolescence to adult psychopathology. The third study in the context of early life stressors investigates resilience, well-being, and mental health behaviors in migrant and non-migrant adolescents tested across six countries (Australia, New Zealand, UK, China, South Africa, and Canada). Authors explore the impact of country-specific factors, migrancy itself, and trauma exposure on resilience, well-being, and mental health among migrant and non-migrant adolescents aged 10–17 showing that migrant adolescents exhibited greater resilience resources than non-migrants despite a higher trauma load (Gatt et al.).

Another group of articles discuss resilience mechanisms in the brain. In the study by Dimitrov et al. resting state fMRI data were acquired before and immediately after stress induction inside the MRI scanner to determine amygdala RSFC. Changes in resting state connectivity from pre- to post-stress were compared between cortisol responders and non-responders. Only non-responding males showed an increased functional connectivity between the posterior cingulate cortex and bilateral amygdala, potentially indicating an increased engagement of the amygdala within the DMN directly after stress in cortisol non-responders. In a review Escamilla et al. discuss the current literature on lifespan-related reorganization processes in the brain that can be related to protective mechanisms that help coping with age-related situations and reduce the burden for brain alterations linked to mental disorders. The authors conclude that correct adaptation of brain connectivity patterns could be the key to healthy aging and diminish the risk for developing neurological and neuropsychiatric disease at different stages across the lifespan. Another review by Bolsinger et al. comprehensively synthesized the literature neuroimaging correlates of resilience to traumatic events, especially focusing on functional connectivity (connectivity of the amygdala, the anterior cingulate cortex, and the prefrontal cortex). Increased prefrontal cortex activity came up to be a protective factor in this study. Finally, increased amygdala and anterior cingulate cortex activities and decreased prefrontal cortex activity as a response to external stimuli were also associated with higher vulnerability, while increased prefrontal cortex activity was associated with lower vulnerability.

Two other studies summarize and evaluate the current state of scientific affairs on the biological basis of resilience and potential interventions. Snijders et al. provide an overview of the literature on animal and human studies of resilience in order to promote resilience. In this review a variety of interventions to promote resilience are proposed such as installing positive emotions and improving cognitive abilities, social interactions, and feelings of purpose and meaning of life along with physical health. Another study by Ben-Zion et al. provides insights into the effects of traumatic events and the mediating factors of cognitive flexibility based on two empirical studies. Cognitive flexibility, shortly after trauma exposure, emerged as a significant predictor of PTSD symptom severity. The findings of these studies shed light on the underlying neurocognitive mechanisms of PTSD symptoms, and demonstrate the effectiveness of an early neurocognitive intervention in relieving PTSD symptoms. Such findings may help develop early mechanism-driven, stage-specific interventions for PTSD, thus improving life resilience.

A crucial novel perspective, which has emerged in resilience studies, is that resilient animals have active adaptive mechanisms that are distinct from actions that reverse deleterious effects in susceptible animals. Therefore, the research aimed in this Research Topic investigates both, the relationship and essential distinction of reversing deleterious effects and ways to cultivate active adaptive mechanisms. An integrated combination of studies from different fields including a wide array of disciplines such as epidemiology, psychiatry, and neurobiology studies will be essential for generating a clearer picture of resilience. Additionally, further studies should focus not only on the resilience of individuals or small human/animal populations, but also communities and societies, which are under considerable pressure at the moment due to the COVID-19 pandemic.

Finally all authors suggest that longitudinal studies are recommended as a pathway to elucidating the factors, and mechanisms and of resilience. Understanding resilient individuals better is a key precursor to the development of interventions and strategies to enhance resilience in the wider population. Taken as a whole, two key messages resonated from the articles included in this Research Topic. Firstly, resilience is a dynamic trajectory, which needs interdisciplinary longitudinal research designs. Secondly, resilience research can contribute to develop neurobehavioral based and justified interventions.

This Research Topic has contributed to the extant literature, not just bringing together most recent research but researchers from different fields of expertise by posing new questions to advance the research conceptually, methodologically, and finally in terms of practice impact. Beyond the intra-individual level, the other levels of resilience (i.e., family, community, and societies) and their interactions need much more dedicated research.

## Author Contributions

JL and OT co-wrote the editorial. All authors contributed to the article and approved the submitted version.

## Conflict of Interest

The authors declare that the research was conducted in the absence of any commercial or financial relationships that could be construed as a potential conflict of interest.

